# Repetitive Calf-Raise Pulse Provocative Test for Popliteal Artery Entrapment Syndrome

**DOI:** 10.7759/cureus.74315

**Published:** 2024-11-23

**Authors:** Salman Qayum, Rohan LNU, Muhammad Haider Baber Ali, Soo Yit Gustin Mak, Anand Pillai

**Affiliations:** 1 Ortho-Geriatrics, Wythenshawe Hospital, Manchester, GBR; 2 Trauma and Orthopaedics, Wythenshawe Hospital, Manchester, GBR; 3 Ear, Nose, and Throat (ENT), Wythenshawe Hospital, Manchester University NHS Foundation Trust, Manchester, GBR; 4 Sports and Exercise Medicine, Wythenshawe Hospital, Manchester, GBR; 5 Trauma and Orthopaedics, Wythenshawe Hospital, Manchester University NHS Foundation Trust, Manchester, GBR

**Keywords:** clinical diagnosis, paes screening, physical examination, popliteal artery entrapment syndrome, provocative test, repetitive calf raises

## Abstract

Popliteal artery entrapment syndrome (PAES) is a rare cause of exertional leg pain in young adults, which is caused by compression of the popliteal artery by the surrounding muscular structure. Due to significant overlap in symptoms with other conditions, limitations of diagnostic imaging, and lack of awareness, PAES is frequently misdiagnosed, resulting in late complications and poor prognosis. Clinical assessment is crucial in making the initial diagnosis and referring for relevant investigations for PAES. In this case report, we have discussed the utility of the repetitive calf-raise pulse (RCRP) test as an easy, accessible bedside provocative test that can be used to diagnose PAES during the initial assessment. The RCRP test, which involves repetitive raises followed by an examination of distal pulses, has not been studied properly, and there is very scant literature regarding this. However, it follows the same underlying principles as Doppler with provocative manoeuvres. We have described a new and novel test for screening PAES, and the adoption of this test for people presenting with exertional leg pain can minimise the likelihood of misdiagnosis by referring selected patients to the appropriate diagnostic pathway.

## Introduction

Popliteal artery entrapment syndrome (PAES) is a low-prevalence pathology that is caused by compression of a popliteal artery by surrounding popliteal fossa myofascial structures. Prevalence ranges from 0.16% to 3.5% [1]. The true incidence of this condition is not known due to difficulties associated with its diagnosis. It was initially described by a medical student, Anderson Stuart, in 1879, and Haming later identified the management in 1971. In the past few years, new diagnostic techniques using duplex ultrasound, computed tomography angiography (CTA), and magnetic resonance angiography (MRA), along with increased awareness of the presenting symptoms, have enhanced the sensitivity in detecting PAES.

Patients with PAES typically present with pain in the feet and the calves on exertion. Symptoms settle down with rest and can be associated with paresthesia, coldness, and cramps. The presentation varies depending on the chronicity of the disease. Late presentation can include calf swelling and pain at rest due to permanent vascular injury or, in severe cases, acute limb ischaemia due to arterial occlusion/thromboembolism [1]. It is not possible to distinguish the anatomic and functional variants on physical symptoms and examination alone, and therefore, further investigations are required to confirm the diagnosis [2].

Duplex ultrasound is normally used as the first line of investigation to diagnose the condition. It is normally done with provocative manoeuvres such as plantar flexion. However, one limitation of ultrasound is the high false-positive rate and reduced sensitivity in diagnosing, especially functional variants [3]. CTA and MRA are now considered the primary diagnostic investigations, which can help differentiate between anatomic and functional variants.

PAES is a hugely underdiagnosed condition due to the significant overlap of symptoms with similar conditions and a high false negative rate of diagnostic imaging. Currently, a combination of imaging investigations is used to diagnose PAES and differentiate it into anatomic and functional variants. There is very limited literature regarding basic clinical manoeuvres as possible provocative tests for PAES. We have reviewed the literature in view of utilising the repetitive calf-raise pulse (RCRP) test as a basic, cost-effective method in screening for PAES in young adults presenting with exertional leg pain in a clinical setting. We also present a case where a patient presenting with exertional leg was assessed using the RCRP test and eventually diagnosed with PAES on ultrasound.

## Case presentation

An 18-year-old gentleman presented in the Orthopaedic clinic with increasing bilateral calf pain for the last four months. The pain initially started following a 5 km run, necessitating him to stop and rest until the pain subsided. He described it as a dull ache in both calves. There was no associated leg numbness, weakness, or pain at rest and no history of injury. He did not report any other aggravating factors for his pain. He used to play four hours of football, which had now been reduced to only one hour per week due to pain. The pain was mostly aggravated by sustained running. At the time of review in the Orthopaedic clinic, his symptoms had worsened, and he reported pain after running for 10 minutes. He was otherwise well in himself with no regular medication or significant past medical history. He had no history of smoking.

On examination, he stood in a pes cavus foot position with a raised longitudinal arch. There was a full range of movement at the hip, knee, and ankle joints bilaterally. There was no tenderness of calf muscles. The dorsalis pedis and posterior tibial pulses were easily palpable on the foot and ankle at rest. The RCRP test was conducted in the clinic, which involved repetitive calf raises in a standing position until the pain was reproduced, and distal pulses were then examined. The pain was reproduced after he did 25 single-leg calf raises on his left leg, and the pulses, including dorsalis pedis and posterior tibialis, were checked immediately afterwards and found to be absent for about a minute. There were no associated skin changes noted. He was referred for a Doppler scan due to suspicion of PAES.

**Figure 1:**
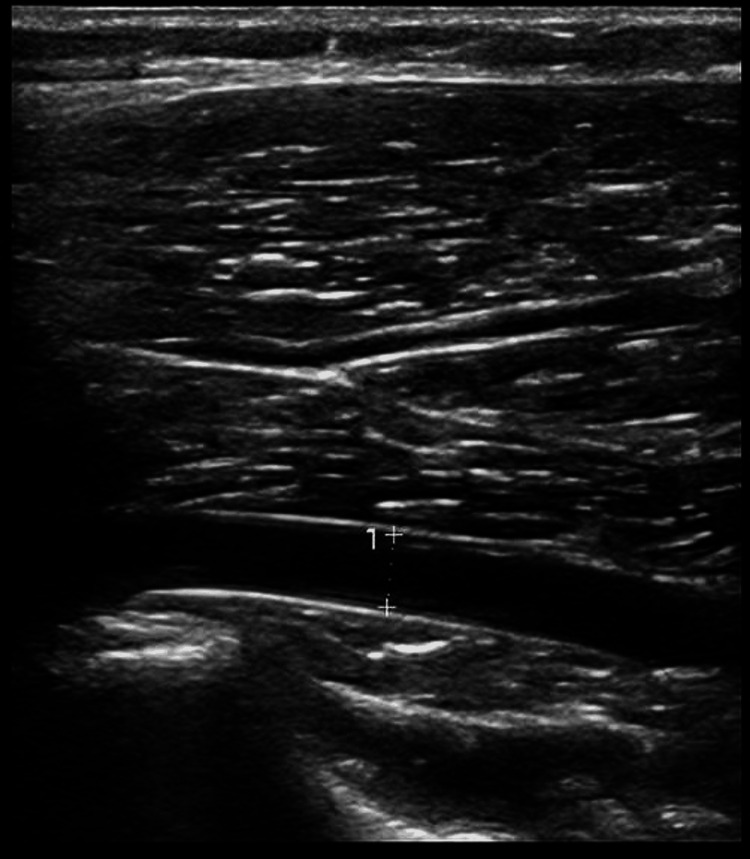
Popliteal artery without provocation Permission obtained from the patient

**Figure 2:**
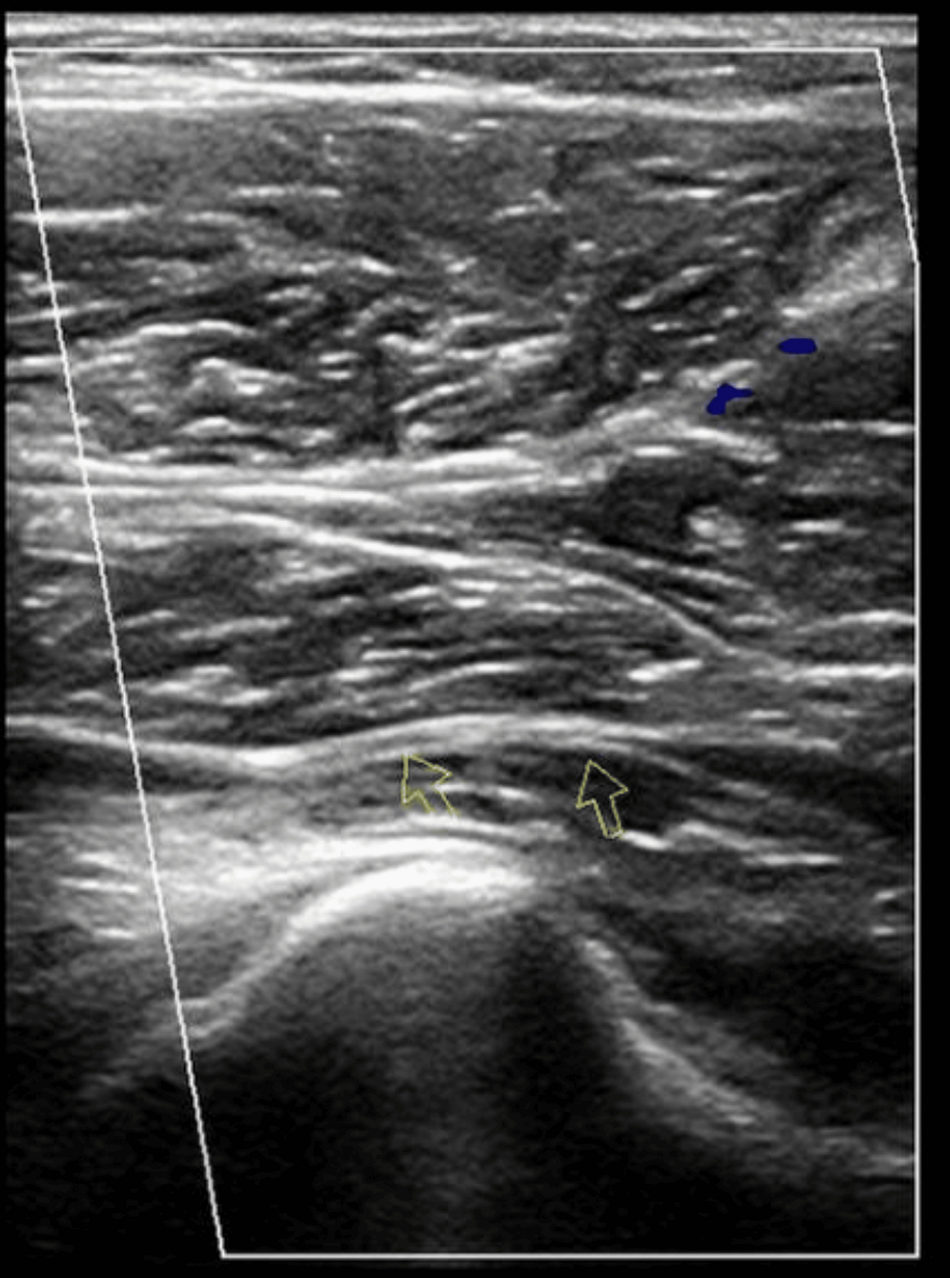
Popliteal artery occluded with plantar flexion Permission obtained from the patient

The US Doppler showed good biphasic flow in both popliteal arteries at rest (Figure 1). On plantar flexion, the right distal popliteal artery was completely occluded in the prone position and widely patent on dorsiflexion. Similarly, the left popliteal artery became fully occluded on plantar flexion and became patent on dorsiflexion with biphasic waveforms (Figure 2). This confirmed bilateral popliteal artery entrapment. The patient was advised to do cycling and gentle strength training. He was referred to vascular surgery for further investigations and management.

## Discussion

Incidence of PAES and clinical assessment

**Figure 3:**
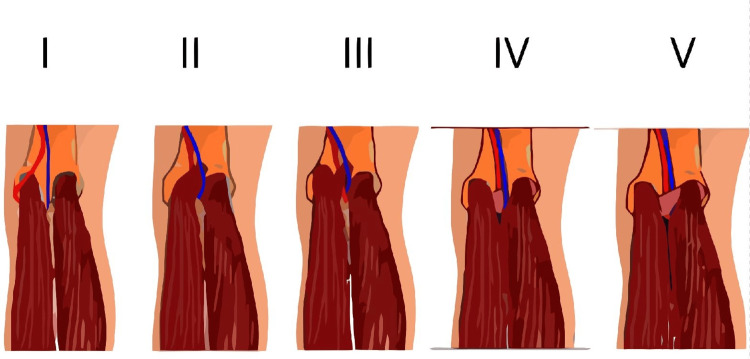
Anatomic classification of popliteal artery entrapment syndrome (PAES) Image credits: Salman Qayum

PAES is a relatively rare cause of exertional leg pain in young people, which is caused by external compression on the popliteal artery by surrounding structures in the popliteal fossa. PAES is broadly divided into two categories: anatomic/congenital and functional. The anatomic variant is caused by abnormal development of the popliteal artery and surrounding muscles and soft tissues. The Rich Classification (Figure 3) is widely used to classify PAES into five different anatomic types based on the abnormal course of the popliteal artery in relation to surrounding structures and the cause of pressure on the popliteal artery. The functional variant is often classified as Type 6 and is caused by hypertrophy of surrounding muscles, including gastrocnemius and soleus, without any anatomic abnormality. It is considered much more common than the anatomic variant and more prevalent in athletes and young adults [1]. Functional PAES is a poorly understood cause of PAES and is frequently underdiagnosed.

Early diagnosis is essential to prevent irreversible, progressive popliteal artery injury, which can eventually lead to arterial occlusion, thrombosis, acute limb ischaemia, or aneurysm formation [4]. Misdiagnosis is common due to lack of typical symptoms and, more commonly, due to overlap in presentation with other conditions causing exertional leg pain. This can lead to the progression of disease and poor outcomes. The median delay of diagnosis of PAES has been reported up to 12 months, with some cases taking as long as 15 years to be diagnosed [4,5].

The effects of these delays on outcomes stress the need for a more streamlined diagnostic pathway in patients younger than 50 presenting with exertional leg pain. The standard diagnostic algorithm for most patients presenting with typical symptoms of PAES includes initial evaluation with a duplex ultrasound with or without the provocative manoeuvres. This is normally followed by CTA or MRA, which can help differentiate between the anatomic and functional types of disease when done with provocation and determine the extent of the disease [6]. Given the importance of early diagnosis of PAES, we suggest using an RCRP test when examining patients with exertional leg pain, as mentioned in the case above. This includes calf raises followed by pulse examination, which can be used as a basic provocative test in addition to history in a clinical setting. This is a cost-effective bedside examination, which can help to diagnose patients early in the course of the disease.

A review by Pandya et al. in 2019 [7] on diagnostic approaches for functional PAES has recommended provocative tests such as plantar flexion during imaging as essential in diagnosing the functional variants of PAES. The case presented in this review examined the posterior tibial and dorsalis pedis, which disappeared with plantar flexion/calf raises. It also suggests using an ankle brachial pressure index (ABPI) and duplex scan as initial investigations in the evaluation of patients suspected of having PAES. The RCRP test follows the same basic principles as a Doppler with provocative manoeuvres, which is normally recommended as an initial investigation for PAES. Provocative manoeuvres such as plantar flexion or positional change are done during a Doppler scan to look for any change in the blood flow. Similar provocative manoeuvres are used during CTA, which shows the importance of provocative tests when diagnosing PAES. The RCRP test replaces the Doppler probe with the palpation method to assess for the distal pulses, which, although not as accurate, can help in making an initial diagnosis and referring the patient to the correct pathway for further imaging.

A review by Hislop et al. in 2014 [2] suggested a clinical test for the initial examination involving 15-20 repetitions of single-leg heel raises followed by palpation of the distal pulses. The onset of exertional leg pain symptoms, coupled with a reduction in pulses or auscultation of bruit immediately following the test, could indicate underlying PAES. The study recommends this test for the initial assessment before the patient is referred for a Doppler scan.

We have identified at least three reports [8-10] when reviewing the literature that mentioned using clinical provocative tests on the initial assessment of patients who were later found to be positive for PAES on imaging. All of these case reports recommend the use of provocative tests as part of the examination in the early stages of the disease. Numerous other studies have reported using Doppler with provocation, resulting in a reduction in blood flow due to popliteal occlusion. A French article by Couzan et al. [11] studied a similar provocative test in 10 patients over the course of three years and compared the results to the results of vascular investigations. They have concluded that the test would be beneficial in diagnosing PAES in the early stages. Similarly, a study by Ghaffarian et al. [12] on the diagnostic algorithm for PAES showed a positive reduction in pedal pulses with provocative manoeuvres in the physical exam in 90% of treated limbs.

Multiple studies in the literature have emphasised the importance of good clinical examination, including provocative tests, to correctly diagnose patients with PAES, who can then be referred for further imaging. Adopting this test as part of the initial assessment would not only reduce the misdiagnosis but could also help with early diagnosis and prevent late complications.

Exertional leg pain differentials

One of the major factors leading to the misdiagnosis of PAES, as mentioned previously, is the overlap in presenting symptoms with other vascular conditions. A case reported by Wady et al. in 2018 [13] presented a 30-year-old female with unilateral burning and pain in the leg who was misdiagnosed with a chronic exertional compartment syndrome (CECS) and underwent fasciotomy. She was eventually diagnosed with PAES 12 years later. The case discussion revealed that she was referred to the CECS pathway at the initial assessment, emphasising the importance of good initial clinical assessment when evaluating these patients. An RCRP test during the initial assessment can help narrow down the differentials of exertional leg pain in young people and place them on the correct pathway in the early stages.

A systematic review by Bosnina et al. in 2023 [14] regarding a diagnostic framework for people presenting with exertional leg pain discussed the nine most common conditions and the differentiating factors on history and examination. The report showed that the key distinctive element for the diagnosis of PAES is the history of intermittent claudication and the positional loss of posterior tibial pulse on examination and with provocation during ultrasound. This would support the use of the RCRP test, which relies on the reduction/absence of pulses following a repetition of calf raises and helps in differentiating it from other conditions.

CECS is one of the differentials that is thought to have significant overlap with PAES in presentation. This makes it challenging to distinguish between the two conditions based on clinical assessment alone. Multiple cases of PAES have been reported to be misdiagnosed as CECS initially, resulting in poor outcomes and delayed diagnosis. A case by Bellomo et al. in 2024 [15] of a patient concurrently diagnosed with CECS and type VI PA discussed the differences in presentation and examination. Imaging has been mentioned as the gold standard for diagnosis; however, one of the key differences in the examination is weaker pedal pulses/ABPI with plantar flexion in PAES compared to CECS. Calf raises/plantar flexion would produce similar changes in pulses and help in differentiating between the two conditions. Similarly, a case report by Gaunder et al. [10] presents a case where a young athlete was misdiagnosed initially and treated for CECS; however, he presented later due to worsening of symptoms and was found to have reduced pulses following plantar flexion on physical examination. The article emphasised the importance of physical examination in the diagnosis of PAES, and it mentioned the reduced pulses after plantar flexion as a 'key examination finding' when differentiating PAES from other conditions.

Treadmill vs. plantar flexion

The treadmill test has been commonly used as part of the initial evaluation in diagnosing vascular diseases. The responses of ABPI to the treadmill test are well-known in lower limb arterial disease or PAES [16]. ABI drops greater than 30% and/or exertional ABI <0.9 have been suggested as a positive result with nearly 100% sensitivity for anatomic PAES [1]. A study by Brown et al. in 2019 [17], which investigated the use of exertional ABPI for diagnosing popliteal artery entrapment, showed a significant decrease in the exertional ABPI one minute after exertion. Patients were assessed after a treadmill test and forceful plantar flexion, and the ABPIs were measured. The results showed a significant drop in ABPI in patients with PAES symptoms compared to asymptomatic patients. Additionally, plantar flexion resulted in popliteal occlusion in 10% of symptomatic patients when assessed with ultrasound.

Even though the plantar flexion performed during this study was not repetitive, this reinforces the idea of using repetitive plantar flexion/calf raises along with vascular examination as a convenient clinical provocative test instead of the more time-consuming treadmill test. We believe the calf raises coupled with pulses examination could act as an excellent alternative to post-exertion ABPI and could guide further investigations to reach a timely diagnosis.

Current utility of the calf-raise test

The calf-raise test is frequently used in sports medicine to assess the properties of muscle-tendon units (MTU). It is commonly used for medical assessment and rehabilitation for injuries such as Achilles tendinopathy [18]. It generally involves repetitive action of planter-flexor muscles in a unipedal stance and is quantified by the number of repetitions performed. A systematic review done in 2009 [19] on the use of calf-raise tests reported that the average normal value of repetitions expected from general populations is 25, while the average for males is 22 and females is 21.

Although most reviews of calf raises have questioned the standardisation of calf-raise tests [18,19], sensitivity and reliability regarding vascular disease have not been properly studied.

In most of the studies reviewed, the suggested diagnostic pathway includes an initial assessment using ABPI/pulse examination with provocation/exercise. The RCRP we suggest and used in the case above is a simple and straightforward bedside provocative test, which could potentially screen most of the PAES. The RCRP test has two components, including the initial calf raises performed by the patient in a standing position for a certain number of raises or until they reproduce the symptoms, which is then followed by immediate palpation of distal pulses, posterior tibial, and dorsalis pedis. The second part of the examination differentiates it from the existing calf-raise test employed by sports physicians, which relies mostly on the number of repetitions and suffers from a lack of standardisation.

Limitations of using the RCRP test

Reviewing the literature, one major limitation of using calf-raise tests to assess diseases is the lack of universal standardisation. As suggested earlier, most sports medicine assessments focus on the number of repetitions before the onset of symptoms. Additionally, several factors can influence the number of repetitions, including age, gender, BMI, and co-morbidities. Factors such as height of raise, pace, and termination criteria also need to be standardised.

Furthermore, the RCRP test is likely to overestimate the diagnosis due to higher false-positive results, which means the normal population might report positive as well [2]. However, further studies and standardisation might help us to improve sensitivity.

The lack of literature discussing the calf raises in patients with vascular disease or its effect on the distal pulses in a normal person is another major constraint when trying to decide its sensitivity as a provocative test.

## Conclusions

PAES is fraught with misdiagnosis due to the overlap of clinical symptoms and diagnostic investigation difficulties. The functional variant, in particular, has significant overlap in symptoms with CECS, as both present with similar neurovascular symptoms on exertion, leading to inappropriate treatment pathways. We propose that the RCRP test can play a vital role in assessing patients who present with exertional leg pain at a primary health centre or emergency department. The test can be carried out easily by the primary physicians or emergency doctors at the bedside without any special training, expertise, or equipment required. When used in conjunction with a thorough clinical history, this would not only reduce the misdiagnosis rates but also prevent delayed diagnosis by facilitating timely referral for a duplex scan. Furthermore, this could have a significant positive financial impact by reducing the financial burden associated with misdiagnosis and complications due to late diagnosis.

One of the drawbacks of using this test is the possibility of false positives, which indicates that it might be more useful for making a diagnosis rather than excluding one. The lack of literature was the major challenge when looking for evidence to support this test as a screening tool. Larger, focused studies would help to establish the RCRP test as a reliable, cost-effective screening tool for PAES, which could help reduce misdiagnosis and improve patient outcomes.
